# Identification and co-evolution pattern of stem cell regulator miR394s and their targets among diverse plant species

**DOI:** 10.1186/s12862-019-1382-7

**Published:** 2019-02-14

**Authors:** Ashutosh Kumar, Vibhav Gautam, Pramod Kumar, Shalini Mukherjee, Swati Verma, Ananda K. Sarkar

**Affiliations:** National Institute of Plant Genome Research, Aruna Asaf Ali Marg, New Delhi, 110067 India

**Keywords:** miR394, *LCR*, Small RNA, Phylogenetic analysis, miRNA evolution

## Abstract

**Background:**

Micro RNAs (miRNAs), a class of small non-coding RNAs, have been implicated in various aspects of plant development. miR394 is required for shoot apical meristem organization, stem cell maintenance and abiotic stress responses in *Arabidopsis,* where it functions by negatively regulating the transcript level of target *LEAF CURLING RESPONSIVENESS* (*LCR*), which is an *F-box* protein-coding gene. The evolutionary conservation of stem cell regulatory miR394-*LCR* module among plants remains elusive.

**Results:**

Our study has identified 79 miR394 and 43 target sequences across 40 plant species using various homology based search tools and databases, and analysed their co-evolution pattern. We customised an annotation workflow which computationally validates 20 novel miR394s from 14 plant species. Independent phylogenetic trees were reconstructed with precursor *MIR394s*, mature miR394s, and their target sequences along with complementary miR394 binding sites. The phylogeny revealed that mature sequences of miR394s as well as their targets belonging to the *F-box* protein encoding gene families, were highly conserved. Though, miR394–3p were complementary to miR394s/miR394–5p, they clustered separately.

**Conclusion:**

The existence and separate clustering of miR394–3p and miR394s/miR394–5p indicate their independent regulation. The phylogeny also suggests that miR394s had evolved at the beginning of gymnosperm-angiosperm divergence. Despite strong conservation, some level of sequence variation in miR394s and the complementary binding sites of their targets suggests possible functional diversification of miR394-*LCR* mediated stem cell regulation in plants.

**Electronic supplementary material:**

The online version of this article (10.1186/s12862-019-1382-7) contains supplementary material, which is available to authorized users.

## Background

MicroRNAs (miRNAs), a class of small non-coding RNAs, have been reported to regulate various aspects of plant development and physiological processes [1–4]. miRNAs function by negatively regulating their target genes mostly through cleavage at mRNA complementary site or by translational inhibition [1, 4, 5]. Often, a family of similar small (21 to 24 nucleotides) functional mature miRNA(s) may be produced through the processing of a single or multiple precursor *MIRNAs* (relatively longer) of the same family gene(s) [1, 6, 7]. Novel miRNAs evolve through events like gene duplication and de novo emergence from different genomic locations and repress specific genes. Young miRNA families have a high evolutionary rate as compared to the primitive ones [8]. A comprehensive understanding of the origin and functional divergence/convergence of these small RNAs can provide cues about the evolution of their target preference.

Unlike protein coding genes, the mature miRNAs and their target complementary mRNA sequences are critical in deciding the functional diversification. Sequence variation in mature miRNA or its complementary sequences may lead to the functional diversification of miRNA mediated gene regulation and its developmental or physiological outcome [9]. Moreover, the miRNA-target interactions are highly conserved, though some studies have predicted and validated non-conserved targets of miR166 and miR167 [9–11]. miR165/166, which generally negatively regulates the transcripts of *HD-ZIPIII* family in a conserved manner, was also shown by us to target *non-HD-ZIPIII* transcripts in *Physcomitrella,* a functional change caused by critical sequence variation [9]. Similarly, we have shown earlier that critical sequence variation in mature miR167 and its complementary binding sites at target mRNA created novel target like *CALCINEURIN B-LIKE10* (*CNBL10*) in apple, besides conserved targets *ARF6*/*8* [10, 11]. This kind of change often leads to the evolution of novel targets, henceforth leading to functional diversification of miRNAs mediated regulation [11].

Unlike animals, the post-embryonic development in plants is uniquely regulated, which is marked by continuous activity of shoot and the root meristems leading to the formation of distinct shoot and root systems [12]. The plant shoot apical meristem (SAM) harbours a self-perpetuating population of pluripotent stem cells and their organizing centre, which are maintained by activity of several key genes and phytohormones [13, 14]. Besides protein coding genes, a few miRNAs, such as miR165/166 and miR394 have been implicated in shoot meristem regulation in *Arabidopsis* [4]. In *Arabidopsis*, the protoderm derived miR394, which are processed from two precursors *MIR394a*/*b*, moves to the distal meristem during embryonic shoot meristem formation by negatively regulating the transcripts of its target *LEAF CURLING RESPONSIVENESS* (*LCR*) which belongs to F-box protein family [6, 15–17]. The miR394-*LCR* regulatory module is also implicated in abiotic stress responses in *Arabidopsis* [18, 19]. Some other F-box protein encoding genes, like *TRANSPORT INHIBITOR RESPONSE1* (*TIR1*) and *MORE AXILLARY GROWTH2* (*MAX2*) are also negatively regulated post-transcriptionally by miR393 and miR528, respectively [16, 20, 21]. Interestingly, miR394 only targets one F-box protein gene *LCR* (At1 g27340) in *Arabidopsis*. Since miR394-*LCR* module play important physiological and developmental role, it is imperative to study its presence and evolutionary pattern among diverse plant species Therefore, a comprehensive analysis of co-evolutionary pattern of miR394 and its targets (*LCR* or non-*LCR*) is required to elucidate the functional conservation and/or divergence of miR394-*LCR*/target module, an important component of stem cell regulation, across plant kingdom. This knowledge may further help in understanding the role of miR394-target in other plant species of interest.

Here we identified and retrieved *MIR394* genes and also predicted, annotated and validated novel miR394s. These were used for the reconstruction of separate phylogenetic trees for precursor and mature sequences along with their respective conserved/non-conserved targets. Our phylogenetic analysis suggests that the mature miR394s were highly conserved than their respective precursor *MIR394* sequences. Subsequently, the phylogeny of identified conserved and non-conserved targets suggested some level of functional diversification, despite strong conservation of miR394-target module in plants. The presence of independent miR394–3p sequences targeting non-conserved genes may also contribute to functional diversification.

## Results

### Identification of precursor and mature sequences of miR394s

miR394 plays a crucial role in the shoot meristem development by regulating the spatial organization of stem cell niches. Therefore, it is imperative to further elucidate the regulatory roles played by miR394s across the plant kingdom. The availability of well annotated complete genome sequences of diverse angiosperms has enabled us to draw genetic relationship between the *MIR394* gene family and their targets across the angiosperms.

**Fig. 1:**
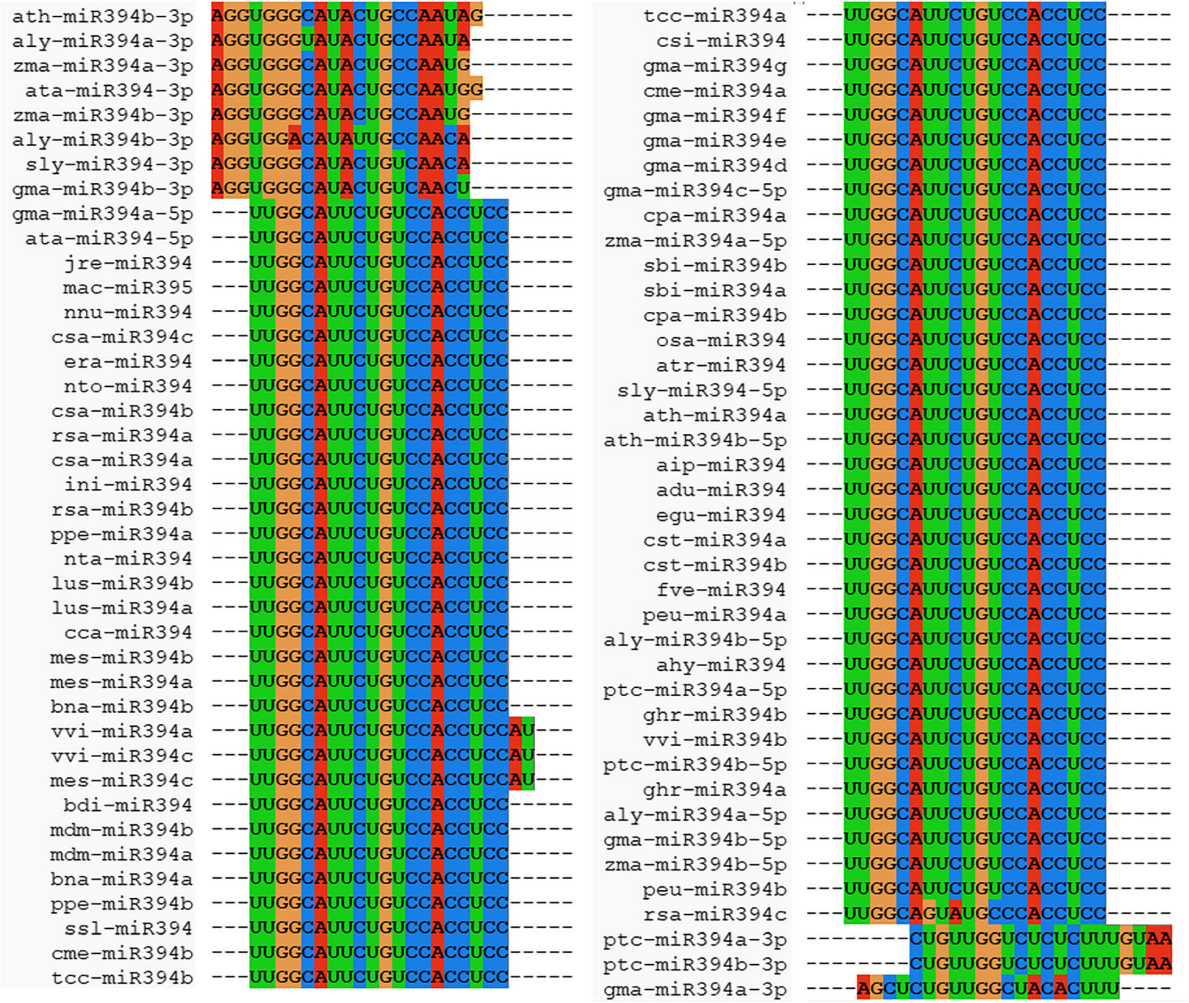
Multiple sequence alignment of retrieved miR394 sequences from miRBase using ClustalX2. The alignment of retrieved 79 miR394 to identify the uniqueness among the sequences based on homology

Though *MIR394* is a small and less studied gene family, we retrieved 59 mature miR394 sequences from 26 different plant species available in miRBase Registry database (http://www.mirbase.org/) (Table 1). Among these 59 sequences, 48 sequences were found to be processed from 5′ end, whereas 11 sequences were processed from 3′ end of the stem-loop precursors (Table 1). The highest number of mature miR394 sequences i.e., 9, were retrieved form the *Glycine max* out of which, 7 were processed from the 5′ end and rest two were processed from the 3′ end of the stem-loop precursor. We however, did not get any homologs of *MIR394* in other plant species except angiosperms. The already registered and newly annotated miR394 sequences were retrieved from the plant species under study and subjected to sequence similarity and uniqueness analysis by multiple sequence alignment (MSA) using ClustalX2 (Fig. 1). The multiple sequence alignment (MSA) showed that most of the mature miR394 sequences share maximum identity except those which were processed from 3′ end of the stem-loop precursors (Fig. 1). The statistical significance of aligned miR394 sequences calculated through Kolmogorov-Smirnov statistical test, suggested that the ≥0.3 fraction of mature miR394 sequences have ≥90% sequence identity (summarized in Additional file 1; Figure S1A). Whereas, ~ 0.2 fraction of the precursor *pre-MIR394* sequences have > 55% sequence identity (summarized in Additional file 1; Figure S1B). Our analysis hereby indicates that in spite of showing a good similarity, the mature miR394s are more conserved than their precursor sequences.

**Table 1:** List of miR394s retrieved from miRBase. Species specific sequences of miR394s retrieved from miRBase. The list contained the number of miR394 which were specifically processed either from 5′ end (miR394/miR394–5p) or 3′ end (miR394–3p) of stem loop precursors

miRBase IDs	Name of plant species	No. of miR394/miR394–5p	No. of miR394–3p	Total No of miR394s
ahy-miR394	*Arachis hypogaea*	1	0	1
aly-miR394	*Arabidopsis lyrata*	2	2	4
ata-miR394	*Aegilops tauschii*	1	1	2
ath-miR394	*Arabidopsis thaliana*	2	1	3
atr-miR394	*Amborella trichopoda*	1	0	1
bdi-miR394	*Brachypodium distachyon*	1	0	1
bna-miR394	*Brassica napus*	2	0	2
cca-miR394	*Cynara cardunculus*	1	0	1
cme-miR394	*Cucumis melo*	2	0	2
cpa-miR394	*Carica papaya*	2	0	2
csi-miR394	*Citrus sinensis*	1	0	1
ghr-miR394	*Gossypium hirsutum*	2	0	2
gma-miR394	*Glycine max*	7	2	9
lus-miR394	*Linum usitatissimum*	2	0	2
mdm-miR394	*Malus domestica*	2	0	2
mes-miR394	*Manihot esculenta*	2	0	2
nta-miR394	*Nicotiana tabacum*	1	0	1
osa-miR394	*Oryza sativa*	1	0	1
ppe-miR394	*Prunus persica*	2	0	2
ptc-miR394	*Populus trichocarpa*	2	2	4
sbi-miR394	*Sorghum bicolor*	2	0	2
sly-miR394	*Solanum lycopersicum*	1	1	2
ssl-miR394	*Salvia sclarea*	1	0	1
tcc-miR394	*Theobroma cacao*	2	0	2
vvi-miR394	*Vitis vinifera*	3	0	3
zma-miR394	*Zea mays*	2	2	4
Total		48	11	59

### The newly annotated *MIR394* family members

**Fig. 2:**
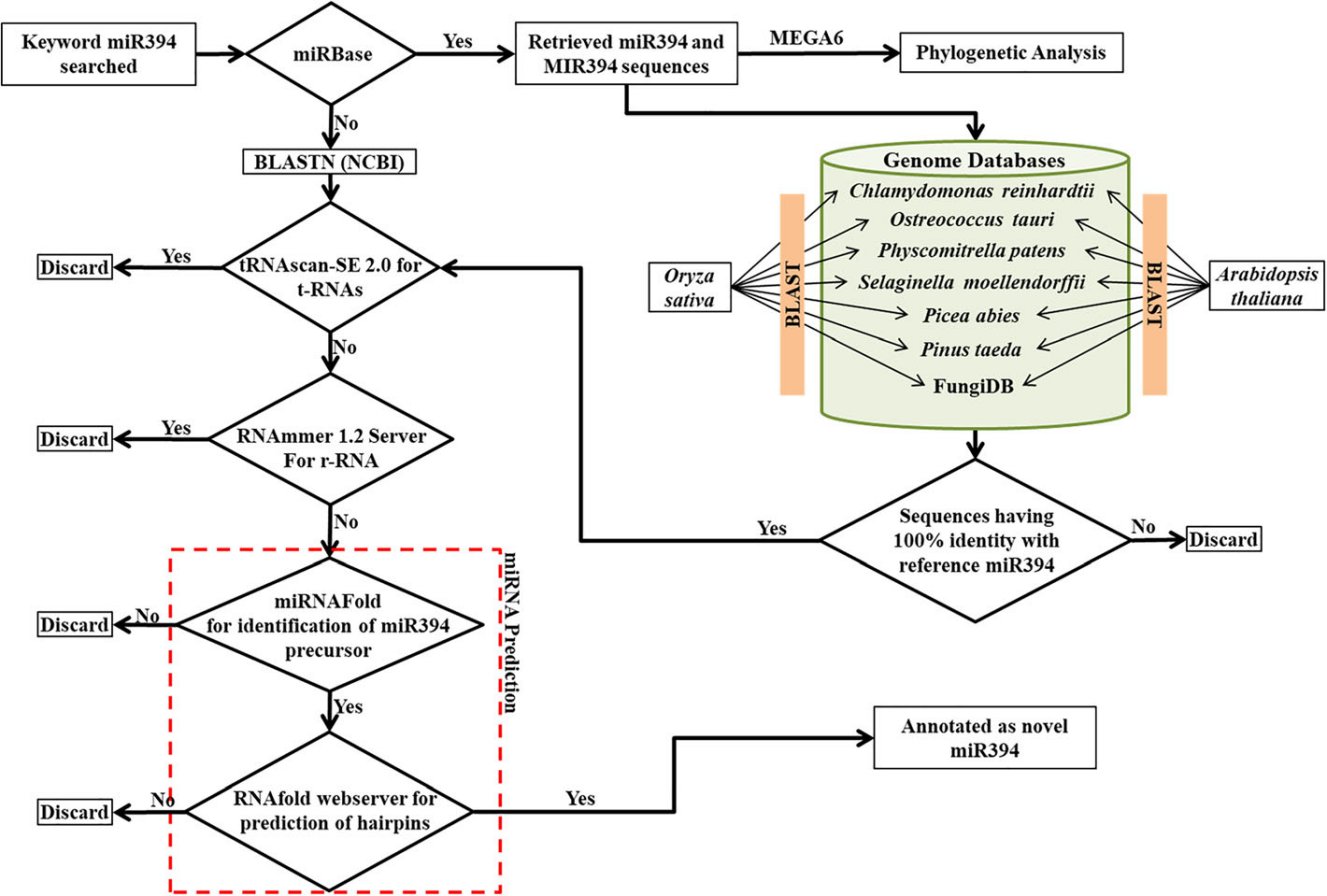
Flow chart for the prediction of new *MIR394* sequences from newly sequenced genome. The known miR394 sequences used to identify and new one using NCBI BLAST. Identified *MIR394* sequences further screened for the prediction of possible stem-loop precursor and annotated using different bioinformatics tools

In the past few years, the advent of sequencing technologies has led to the availability of whole genome sequences of a number of plant species in public databases. Apart from miR394s sequences registered in miRBase, we also identified some more homologs of *MIR394* sequences from the newly sequenced genomes available in the NCBI database. These homologs of *MIR394*, were subjected to a computational workflow which involved the prediction, in silico validation, and annotation of miR394 sequence structures for the plant species under study. The flowchart (Fig. 2) for the aforesaid computational workflow has been explained in the materials and methods section. A total of 20 new miR394s stem-loop precursors were validated in silico from 14 different species (Table 2). We identified three new paralog sequences of *MIR394* from each *Camelina sativa* and *Raphanus sativus*. Similarly, two new paralog sequences of *MIR394* were identified from each of *Eucalyptus grandis*, *Cucumis sativus* and *Populus euphratica* (Table 2). These findings about the new members of *MIR394* family might be helpful in understanding their functional conservation/divergence.

**Table 2:** List of putative miR394s predicted from newly sequenced genome. The *MIR394* sequences from newly sequenced genome were identified through our customized workflow (Fig. 2) and predicted using miRNAFold tool citing their co-ordinates. The putative miR394s of respective *MIR394* were validated using RNAfold webserver, which calculates minimum free energy (MFE) of the canonical secondary structure of predicted miRNAs. The nomenclature of the predicted miRNAs was in accordance with miRBase nomenclature system

Name of plant species	Gene Id	miR394 IDs	Predicted Precursor from *MIR394*		Predicted minimum free energy (kcal/mol)	Putative miR394 Sequence
			Start Position	End Position		
*Arachis duranensis*	LOC107481607	adu-miR394	314	467	−63.37	UUGGCAUUCUGUCCACCUCC
*Arachis ipaensis*	LOC107634751	aip-miR394	340	495	−71.4	UUGGCAUUCUGUCCACCUCC
*Camelina sativa*	LOC104740735	csa-miR394a	509	666	− 77.2	UUGGCAUUCUGUCCACCUCC
*Camelina sativa*	LOC104756391	csa-miR394b	408	555	−70.7	UUGGCAUUCUGUCCACCUCC
*Camelina sativa*	LOC104702369	csa-miR394c	544	692	−58.2	UUGGCAUUCUGUCCACCUCC
*Cucumis sativus*	LOC105435326	cst-miR394a	285	429	−51.9	UUGGCAUUCUGUCCACCUCC
*Cucumis sativus*	LOC105434629	cst-miR394b	196	350	−53.9	UUGGCAUUCUGUCCACCUCC
*Erythranthe guttatus*	LOC105950700	egu-miR394	248	407	−53.3	UUGGCAUUCUGUCCACCUCC
*Eucalyptus grandis*	LOC104450295	era-miR394	213	370	−52	UUGGCAUUCUGUCCACCUCC
*Fragaria vesca*	LOC105349875	fve-miR394	538	675	−57.5	UUGGCAUUCUGUCCACCUCC
*Ipomoea nil*	LOC109181078	ini-miR394	524	681	−67.1	UUGGCAUUCUGUCCACCUCC
*Juglans regia*	LOC108984513	jre-miR394	507	663	−64.8	UUGGCAUUCUGUCCACCUCC
*Musa acuminata*	LOC108951711	mac-miR394	155	327	−73.6	UUGGCAUUCUGUCCACCUCC
*Nelumbo nucifera*	LOC109115150	nnu-miR394	562	718	−46.1	UUGGCAUUCUGUCCACCUCC
*Nicotiana tomentosiformis*	LOC104110977	nto-miR394	188	326	−49.3	UUGGCAUUCUGUCCACCUCC
*Populus euphratica*	LOC105109932	peu-miR394a	1175	1323	−70.7	UUGGCAUUCUGUCCACCUCC
*Raphanus sativus*	LOC108816492	rsa-miR394a	108	261	−58.5	UUGGCAUUCUGUCCACCUCC
*Raphanus sativus*	LOC108816348	rsa-miR394b	117	270	−57.4	UUGGCAUUCUGUCCACCUCC
*Raphanus sativus*	LOC108816010	rsa-miR394c	1742	1842	−40	UUGGCAGUAUGCCCACCUCC
*Populus euphratica*	LOC105137284	peu-miR394b	1196	1344	−65	UUGGCAUUCUGUCCACCUCC

### miR394s/miR394–5p sequences are more conserved than miR394–3p

**Fig. 3:**
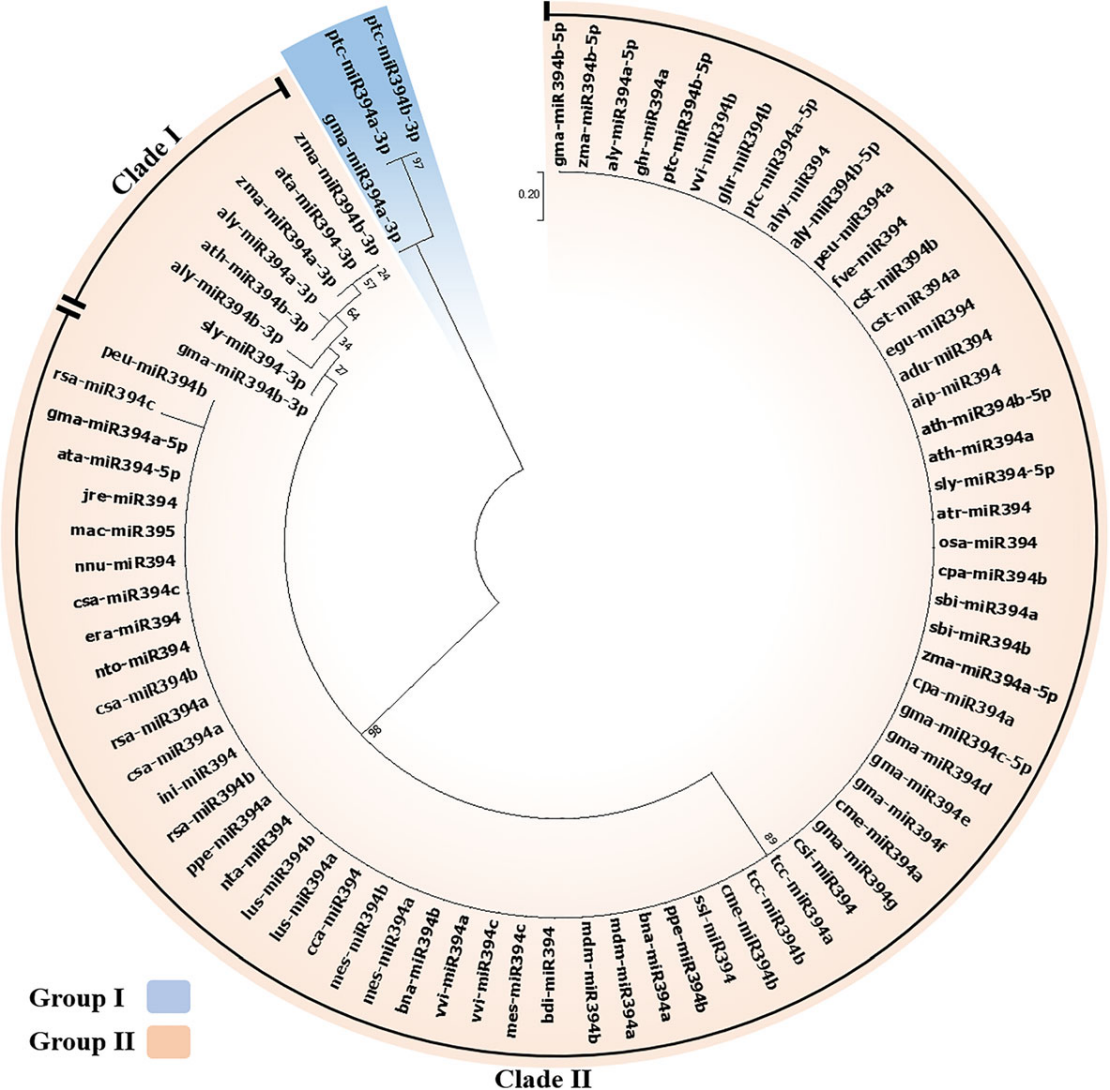
An unrooted ML phylogeny of miR394s using MEGA6. The tree is divided into two groups group I (represented in blue colour) and group II (represented in pink colour). The group I contains miR394–3p (processed from 3′ end of precursor) and group II have miR394/miR394–5p sequences (processed from 5′ end of precursor) sequences, also sub-divided into clade I and II. The scale bar represents the nucleotide substitution rate

The phylogenetic study carried out for revealing the genetic relationship between *MIR394* genes and mature miR394s sequences across the plant kingdom were completed using Neighbour-Joining (NJ) and Maximum Likelihood (ML) methods, respectively, with parameters described later. The ML tree for 79 mature miR394 sequences branched off into two groups (group I and II) with high bootstrap value (Fig. 3). The group I had the smallest cluster consisting only of three sequences, whereas group II contained rest of the miR394 sequences. Further, group II was divided into two clades, clades I and II (Fig. 3). The ML tree showed that the group I, and clade I from group II had all the miR394 sequences processed from the 3′ end (miR394–3p). The clade II of group II had only miR394 sequences which were processed from 5′ end (miR394s/miR394–5p). Interestingly, it was observed that the clusters containing miR394–3p sequences had diverged randomly with varied branch lengths (group I and clade I of group II), however clade II (group II) showed a uniform branch length. Group I in the ML tree had only three sequences ptc-miR394a-3p, ptc-miR394b-3p and gma-miR394a-3p. The ptc-miR394a-3p and ptc-miR394b-3p diverged from the gma-miR394a-3p (Table 1) with a higher rate of substitution, evident from its longer branch length (Fig. 3). Clade I from group II contained the sequences which separated from gma-miR394b-3p. Similar to group I, all the miR394–3p sequences randomly diverged with varied rate of substitution (Fig. 3). The first separation from common ancestor in clade I was shown by sly-miR394–3p followed by aly-miR394b-3p sequence (Table 1). The next separation formed two clusters, one had the sequences zma-miR394a/b-3p and ata-miR394–3p, and the second had aly-miR394a-3p and ath-miR394b-3p (separated with higher rate of substitution) (Table 1). These three sequences, zma-miR394a/b-3p and ata-miR394–3p, separated with a uniform substitution rate (Fig. 3). Interestingly, clade II in ML tree contained miR394s/miR394–5p sequences, which were uniformly placed with a constant rate of substitution and high bootstrap value, hence signifying a high conservation of these sequences (Fig. 3). The nomenclature of “miR394–3p”, signifies that these sequences are processed from 3′ end of stem loop precursors, complementary to miR394s/miR394–5p. Therefore, a separate ML tree was reconstructed using reverse complementary sequences of miR394–3 ps (miR394–3pRC) along with other miR394s (summarized in Additional file 1; Figure S2). Interestingly, the ML phylogeny revealed that miR394–3pRC along with miR395-3p sequences clustered separately (summarized in Additional file 1; Figure S2) similar to the miR394 ML tree (Fig. 3). Here, all the miR394–3pRC sequences formed separate cluster, except the sequences ptc-miR394a/b-3pRC and gma-miR394a-3pRC (summarized in Additional file 1; Fig. S2) which distantly clustered from the miR394–3p sequences with the same topology.

### Random divergence was present among *precursor MIR394s* (*pre-MIR394*) sequences

**Fig. 4:**
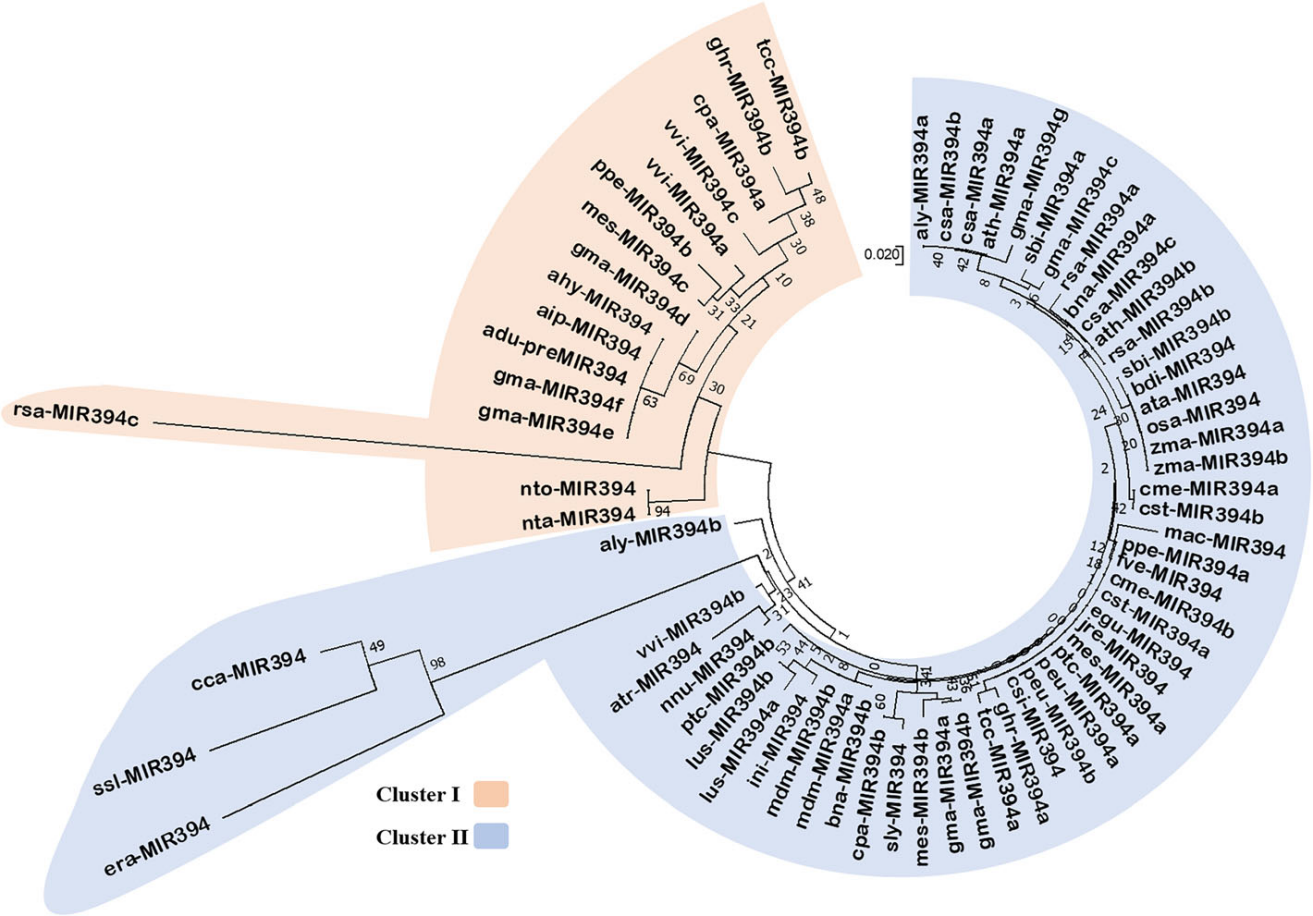
An unrooted NJ phylogeny of *pre-MIR394*s using MEGA6. The tree is sub-divided into cluster I and II represented in pink and blue colour, respectively. The *pre-MIR394* sequences has shown discrete evolution pattern with varying substitution rate. The scale bar represents the nucleotide substitution rate

We also analysed the genetic relationship among *pre-MIR394s* sequences by reconstructing the NJ tree. The NJ tree branched off many times to produce sub-clades with a varied rate of substitution (Fig. 4). All *pre-MIR394s* were divided into two clusters from a common ancestor (Fig. 4). The first separation in the cluster I was shown by closely related *nto-pre-MIR394* and *nta-pre-MIR394* (Table 2). Subsequent separation was of *rsa-pre-MIR394*, which has a longest branch length due to higher substitution rate (Fig. 4, Table 2). In cluster I, rest of the *pre-MIR394s* sub-divided to form two clades with random distribution, variable branch length and substitution rate. In cluster II, the first divergence was of *aly-pre-MIR394* from common ancestor. Further branching placed the clade formed by three *pre-MIR394s* distantly from other sequences with the highest branch length. This clade contained closely related *cca-pre-MIR394* and *ssl-pre-MIR394* which separated from *era-pre-MIR394* (Tables 1 and 2), have evolved fast evident from higher substitution rate (Fig. 4). Rest of the *pre-MIR394s* homologs in cluster II were placed randomly and sub-divided into many sub-clades with varied substitution rates. Interestingly, most of monocot *pre-MIR394s* formed a separate single sub-clade in cluster II. Likewise, most of the newly identified *pre-MIR394s* also formed a separate single sub-clade with a lower substitution rate and very poor bootstrap value (Fig. 4).

Unlike mature miR394 sequences which were found to be highly conserved, the NJ tree formed by *pre-MIR394* sequences showed less conservation which is evident from the formation of multiple branches and clusters, with a very random rate of substitution (Fig. 3). The sequence alignment statistics also supported this randomness among the precursor sequences (summarized in Additional file 1; Figure S1B).

### Unique identical miR394s (UmiR394s) majorly target transcripts belonging to *F-box* gene family

During the course of evolution and selection, many target genes have undergone sequence variation in the binding site. Therefore, it is imperative to understand the uniqueness of miR394 sequences for targeting transcripts of conserved/diverged gene/s, although having variation at their complementary binding site/s. This study will help in elucidating the co-evolution pattern of miR394, their targets and miR394 mediated gene regulation.

The MSA of the miR394 sequences formed different clusters of mature sequences depending upon their sequence similarity and uniqueness. A total of eight unique clusters were formed (UmiR394–1 to 8). Further, we observed that only one cluster (UmiR394–1) was shared by large number of species with only miR394s/miR394–5p sequences, one more cluster was found (UmiR394–2) to contain only miR394s/miR394–5p sequences, shared by two species (Table 3). The MSA result showed that rest of the clusters (UmiR394–3 to 8) contained only miR394–3p sequences, whereas, four clusters (UmiR394–3, UmiR394–5 to 7) were shared by two species and two clusters have only one miR394–3p sequence (Table 3). The largest cluster UmiR394–1 has maximum number of miR394 sequences (65) from 40 different species along with maximum number of 11 Unique Target Sites (UTSs) which were predicted to target transcripts from 38 genes. Further, we observed that two clusters UmiR394–2 and 5 have three miR394 sequences from two species each sharing only one UTS with target genes. The UmiR394–3, 6 and 7 have two miR394 sequences, however UmiR394–6 and 7 each have two miR394 sequences from two species, without any UTS. UmiR394–3 has two miR394 sequences from same species with one UTS targeting one transcript (Table 3).

**Table 3:** Identification of unique miR394 sequences and their predicted targets using *psRNATarget* server. List of Unique miR394s (UmiR394s), their Unique Target Sequences (UTSs) which were complementary binding site of UmiR394s bearing a unique identification number (UR). The UTSs were used for the target identification through *psRNATarget* server. The targets were selected on the basis of E-Value and unpaired energy (UPE) evaluated on the basis of target-site accessibility and mode of inhibition

UmiR394	miRNA Id	Unique miR394 Sequence	UR	Unique TargetSequence	E-value	Target Accessibility (UPE)	Target Accession	Target Description	Inhibition
1	ath-miR394a,ath-miR394b-5p,osa-miR394,sbi-miR394a,b,zma-miR394a-5p,zma-miR394b-5p,ptc-miR394a-5p,ptc-miR394b-5p,vvi-miR394b,ghr-miR394a,b,aly-miR394a-5p,aly-miR394b-5p,ahy-miR394,gma-miR394b-5p,gma-miR394a-5p,gma-miR394c-5p,d,e,f,g,csi-miR394,tcc-miR394a,b,bdi-miR394,ssl-miR394,bna-miR394a,b,mes-miR394a,b,cca-miR394,lus-miR394a,b,nta-miR394,ppe-miR394a,b,mdm-miR394a,b,cme-miR394a,b,cpa-miR394a,b,atr-miR394,sly-miR394–5p,ata-miR394–5p,ini-miR394,csa-miR394a,b,c,nnu-miR394,jre-miR394,mac-miR394,era-miR394,nto-miR394,rsa-miR394a,b,c,aip-miR394,adu-miR394,egu-miR394 cst-miR394a,b,fve-miR394,peu-miR394a,b	UUGGCAUUCUGUCCACCUCC	1a	GGAGGUUGACAGAAUGCCAA	1	14.87	AT1G27340	*Ath-LCR*, LEAF CURLING RESPONSIVENESS	Cleavage
					1	16.78	POPTR_0001s13770g	*Ptc-F-box family protein*	Cleavage
					1	17.38	POPTR_0003s16980g	*Ptc-hypo* (F-box)	Cleavage
					1	16.82	LOC100242085	*Vvi-F-box family protein*	Cleavage
					1	15.82	LOC100800464	*Gma-F-box only protein 6-like* (1)	Cleavage
					1	13.67	LOC100810179	*Gma-F-box only protein 6-like* (2)	Cleavage
					1	15.65	LOC100795488	*Gma-F-box only protein 6-like* (3)	Cleavage
					1	16.47	LOC109152509	*Ini-F-box only protein 6-like* (1)	Cleavage
					1	14.044	LOC109175636	*Ini-F-box only protein 6-like* (2)	Cleavage
					1	14.867	LOC104757055	*Csa-F-box only protein 6-like* (1)	Cleavage
					1	15.009	LOC104741342	*Csa-F-box only protein 6-like* (2)	Cleavage
					1	14.867	LOC104776781	*Csa-F-box only protein 6-like* (3)	Cleavage
					1	17.161	LOC104592877	*Nnu-F-box only protein 6-like* (1)	Cleavage
					1	16.572	LOC104591738	*Nnu-F-box only protein 6-like* (2)	Cleavage
					1	15.37	LOC109003194	*Jre-F-box only protein 6-like* (1)	Cleavage
					1	16.4	LOC104415679	*Era-F-box only protein 6*	Cleavage
					1	17.6	LOC104116922	*Nto-F-box only protein 6*	Cleavage
					1	17.61	LOC108809705	*Rsa-F-box only protein 6*	Cleavage
					1	17.1	LOC101211089	*Cst-F-box only protein 6*	Cleavage
					1	15.55	LOC101312115	*Fve-F-box only protein 6*	Cleavage
			1b	GGAUGUGUGCAGAGUGCCAA	3	15.92	AT3G48460	*Ath-GDSL-motif esterase/acyltransferase/lipase*	Cleavage
			1c	GGAGGAGGACAGAGAUGCCAA	3	24.57	AT5G09670	*Ath-loricrin-related*	Cleavage
			1d	GGAGGUGGAAGAAUGCCGG	3	20.99	AT3G12120	*Ath-ATFAD2*, FAD2, FATTY ACID DESATURASE 2	Translation
			1e	GGAGGUGGACAGAAUGCCAA	0	21.42	Os01 g69940	*Osa-OsFBX32 - F-box domain containing protein*	
					0	23.55	SORBIDRAFT_03g044270	*Sbi-hypo 1* (F-box domain)	Cleavage
					0	23.11	LOC103636344	*Zma-F-box only protein 6-like* (not annotated)	Cleavage
					0	22.96	LOC100193727	*Zma-Uncharacterized1* (F-box only protein 6-like)	Cleavage
					0	22.083	LOC103972833	*Mac-F-box only protein 6*	Cleavage
			1f	GGAGGUCGACAGAAUGCCAA	1	14.89	LOC105962383	*Egu-F-box only protein 6*	Cleavage
			1 g	GGAGUUGGACAGAAUGCAAA	2.5	13.3	Os05g51150	*Osa-RNA polymerase sigma factor*	Cleavage
			1 h	CAAGGUGGACAGAAUGCUAA	2.5	16.14	SORBIDRAFT_02g034550	*Sbi-hypo 2* (Transcription factor GRAS)	Cleavage
			1i	GGAAGUGGACAGAGUGCUGA	2.5	18.13	LOC100242751	*Vvi-PMT18*	Cleavage
			1j	GAAGGUGGACAGAGUGCUAC	3	20.26	LOC100253052	*Vvi-FGGY carbohydrate kinase domain-containing protein*	Cleavage
			1 k	GGAGGUAGACAGAAUGCCAA	1	18.77	LOC109010064	*Jre-F-box only protein 6-like* (2)	Cleavage
					1	14.65	LOC107631986	*Aip-F-box only protein 6*	Cleavage
					1	14.65	LOC107482128	*Adu-F-box only protein 6*	Cleavage
					1	15.63	LOC105133704	*Peu-F-box only protein 6-like* (1)	Cleavage
					1	16.33	LOC105123018	*Peu-F-box only protein 6-like* (2)	Cleavage
2	vvi-miR394a,c,mes-miR394c	UUGGCAUUCUGUCCACCUCCAU	2	AAGGAGGUUGACAGAAUGCCAA	1	16.82	LOC100242085	*Vvi-F-box family protein*	Cleavage
3	ptc-miR394a-3p,ptc-miR394b-3p	CUGUUGGUCUCUCUUUGUAA	3	UUACAAAGAGAGACCAACAG	0	17.4	POPTR_0002s11320g	*Ptc-Hypo1* (DUF1005)	Cleavage
4	gma-miR394a-3p	AGCUCUGUUGGCUACACUUU	4	UGAGUGCAGCCAGCAGAGCU	3	14.74	LOC100306608	*Gma-Unch* (DUF3774)	Cleavage
5	zma-miR394a-3p,zma-miR394b-3p,ata-miR394–3p	AGGUGGGCAUACUGCCAAUG	5	CAUUGGCAGUAUGCCCACCU	0	27.31	LOC100383214	*Zma-Uncharacterized 2*	Cleavage
6	sly-miR394–3p,gma-miR394b-3p	AGGUGGGCAUACUGUCAAC	6	NA	–	–	–	–	–
7	ath-miR394b-3p,aly-miR394a-3p	AGGUGGG[C/U]AUACUGCCAAUA	7	NA	–	–	–	–	–
8	aly-miR394b-3p	AGGUGGACAUAUUGCCAACA	8	CGUUGGUAAUAUGUCUGCCU	2.5	20.27	XM_002894202	*Aly-Hypo* (Pectinesterase inhibitor domain)	Cleavage

The MSA showed clustering of miR394 sequences into eight UmiR394 clusters according to their sequence similarity, which were further used for the identification of transcripts having complementary binding sites of UmiR394s using *psRNAtarget* tool. Many a times, *psRNAtarget* tool did not identify any target for UmiR394s, hence we also used the NCBI BLAST. Altogether, a total of 43 transcripts were identified for UTSs targeted by 8 UmiR394s. Surprisingly, *psRNAtarget* or NCBI BLAST was unable to identify any UTSs for UmiR394–6 and 7 in plant species. We observed that most of the identified target transcripts were homologs of *F-box* protein encoding gene family members and rest were either hypothetical or uncharacterized protein coding homologs. Furthermore, *psRNAtarget* tool predicted the *LCR* in *Arabidopsis* (UTS 1a), a validated target of ath-miR394, along three more different targets (UTS 1b to 1d). The UTS 1a cluster was shown to target maximum (20) numbers of target genes (Table 3). In crops, three targets were predicted in maize (UTS 1e and 5) and *Glycine max* (UTS 1a), two target genes in rice (UTS 1e and 1 g) and *Sorghum bicolor* (UTS 1e and 1 h). The three targets identified in maize were transcripts of genes encoding uncharacterized/unannotated proteins, though two of them have domains of F-box protein (predicted using IntroProScan v5; https://www.ebi.ac.uk/interpro/search/ sequence-search). Likewise, one of the target from rice and *Sorghum bicolor* (predicted using IntroProScan v5) belonged to *F-box* protein encoding gene, whereas all three predicted targets in *Glycine max* were *F-box* protein encoding genes (having three different locus) (Table 3).

### Phylogenetic analysis revealed strong conservation of targets of miR394s

**Fig. 5:**
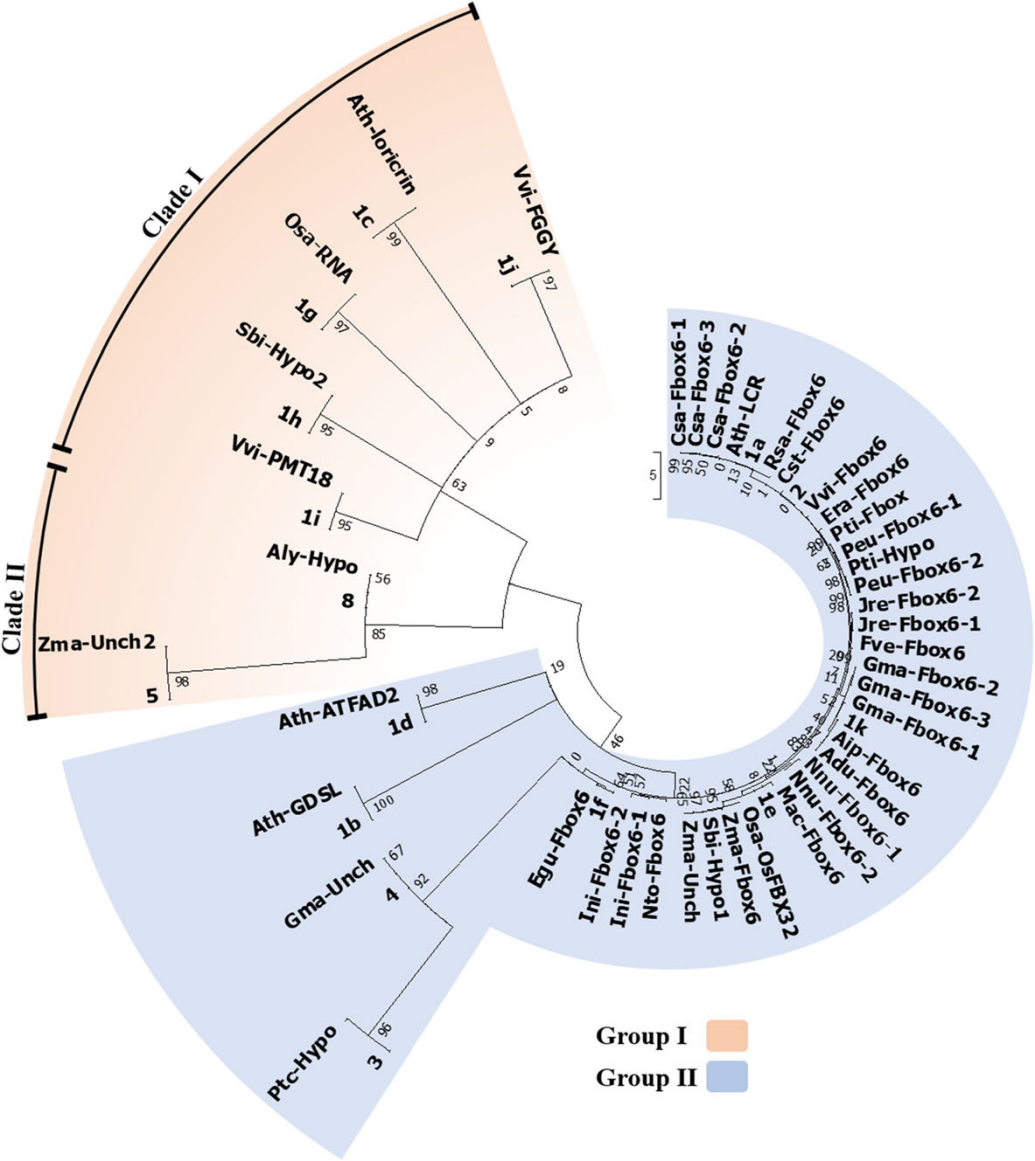
ML Phylogeny of UTSs and their corresponding predicted target genes using MEGA6. The phylogenetic tree is used for the identification of genetic relationship between UTSs of unique miR394 sequences and their targets. The tree is divided into two groups i.e., group I (represented in pink colour) and II (represented in blue colour), further group I is sub-divided into two clades i.e., clade I and II. The target *F-box* homologs are highly conserved. The scale bar represents the nucleotide substitution rate

The miRNAs and their targets are often found to be conserved, although there are reports about divergence of targets. Therefore, to understand the functional conservation/diversification of miR394s, we studied the genetic relationship by reconstructing a phylogenetic tree from the UTSs and their target gene sequences (Fig. 5). The resultant ML tree was divided into two groups; group I and II. Group I further sub-divided into two clades, and the group II was sub-divided into many clusters (Fig. 5). Though clade I has UTSs which were complementary binding sites of the miR394s/miR394–5ps, it mostly contained non-conserved target genes with a higher rate of substitution, whereas clade II has UTS 5 and 8 closely related with *zma-Unch2* and *aly-Hypo* (UTSs which were complementary binding sites of the miR394–3p), respectively (Fig. 5, Table 3).

The first divergence from the ancestor in the group II was of UTS 1d and its closely related non-conserved *ath-ATFAD2* target gene, along with UTS 1b closely related with *ath-GDSL* which is also a non-conserved target gene of miR394 (Fig. 5, Table 3)*.* Subsequent divergence was shown by largest cluster in group II, where the first separation was of UTS 3 and 4 (complementary binding sites of the miR394–3p), closely related with the non-conserved target genes *ptc-Hypo* and *gma-Unch* (Table 1), respectively with higher rate of substitution. The rest of the sequences in the group II contained conserved target genes, i.e., the *F-box* homologs, along with their respective UTSs (complementary binding sites of the miR394s/miR394–5p) which branched off into many sub-clades with lower substitution rate (Fig. 5, Table 3). Some of the conserved targets in the group II were annotated, however most of the target genes were predicted (Fig. 5).

## Discussion

Ubiquitination is a post-translational regulatory process, which controls the activity of numerous proteins during plant growth and development [22]. The F-box proteins are central component of Suppressor of Kinetochore Protein 1(SKP1)-Cullin (CUL)-F-Box complex [23], where the F-box is attached to SKP1 through its highly conserved amino (N)-terminal ‘F-box’ motif, leaving the variable C-terminal to bind with different ligands [24]. About 1600 genes in *Arabidopsis* encode proteins involved in ubiquitination process of which 700 encode F-box subunit [25, 26]. The *LCR,* belongs to one of the members of the *F-box* gene family and largely dictates the involvement of the miR394s in various plant processes [17]. Typically, the miR394s are involved in the regulation of SAM organization, fruit and seed development [27], and regulating the abiotic stress responses in *Arabidopsis* through impairing of pathways involving *LCR* by post-transcriptional modification [15, 16, 19]. In order to understand the diverse function of miR394 regulating *LCR*, we studied their co-evolution using phylogenetic analysis. While retrieving the known precursor and mature sequences of miR394, we observed that miR394 family comprised very less members/isoforms (Table 1) [28]. The search for new *MIR394* homologs from newly sequenced genomes and from other phylum of plant kingdom using our annotation workflow (Fig. 2) [29, 30], revealed a large number of genomes belonging to the different phyla of the plant kingdom which are not yet sequenced. This has created a gap in understanding the evolution of miR394s amongst various plant species. In our study, the retrieved sequences of precursor and mature miR394 belonged to angiosperms, which were used for the reconstruction of phylogenetic tree to identify their genetic relationship and target conservation. Subsequently, the unrooted ML tree of mature miR394s showed less divergence among the miR394 family members across the plant species (Fig. 3). The tree showed two distinct clusters for miR394–3p and miR394/miR394–5p sequences. The cluster of miR394/miR394–5p sequences were found to be highly conserved which was evident from higher homology level and very less substitution rate (due to uniform small branch length in ML tree). This was also evident from the statistical test, Kolmogorov-Smirnov test, which shows ≥0.3 fraction of mature miR394 sequences have ≥90% sequence identity (Additional file 1A). Though miR394–3p sequences formed a separate cluster, they showed a very discrete distribution with higher rate of substitution. This diversification among the miR394–3p sequences might be due to differences in their origin, as they are processed from the 3′ end of stem loop precursors. The miR394–3p sequences are processed from the 3′ end of stem loop precursors but are not the exact complements of miR394/miR394–5p, therefore miR394–3p sequences might have shown variation in sequences. A slight change in the sequence of these 20 bp long miRNAs, may significantly contribute in their divergence. This is also supported by our previous studies on miR167 [10]. The ML tree with reverse complementary sequences of miR394–3p (miR394–3pRC) revealed results similar to the previous phylogeny (Additional file 1; Figure S2; Fig. 3). All the miR394/miR394–5p sequences separated from the common ancestor with a constant rate of substitution (due to higher homology among sequences), whereas the miR394–3p and miR394–3pRC sequences separated with a higher rate of substitution into two clusters. The sequence variation is also evident from the positioning of ptc-mir394a/b-3pRC and gma-miR394–3pRC in the phylogeny (summarized in Additional file 1; Figure S2). These sequences are placed very distantly from their respective miR394–3p sequences, suggesting significant variation between the miR394–3p and miR394/miR394–5p sequences. Additionally, the miR394–3p sequences are processed from the 3′ end (anti-sense) of their precursor sequences, represented as miR394–3p, rather than miR394*. Therefore, we postulated that not all miR394*s are degraded during the processing of precursor, somewhat they function independently as miR394–3p as indicated in the miRBase (http://www.mirbase. org/), similar to our previous study [10]. Conventionally, the stem loop structure of pri-miRNAs is cleaved by DICER-like proteins to produce mature strands either from -5p or -3p, where the mature strand (mature miRNA) is responsible for its ability to enter the RISC complex to bind with target transcripts depicting its functionality, whereas the less stable strand is degraded [31]. However, many reports stated about the prevalence of independent -5p and -3p miRNAs in animal system [32–34], though miRBase reports the presence of both -3p and -5p miRNAs in some families. Further, several conserved miRNA-3ps are identified and reported in animal system [34] the regulatory activities of whom are selected and stabilized by natural selection [8].

The number of miR394 sequences in miRBase repository are from very few species and also very less in number, out of these only one species *Glycine max* has the maximum number of miR394 isoforms (7 miR394s/miR394–5ps and 2 miR394–3ps) (Table 1). All gma-miR394/− 5ps are highly conserved and placed in a single cluster (Fig. 3). Further, the *gma-pre-MIR394* sequences did not group in a single cluster. They showed a discrete pattern of evolution with a varied rate of substitution, scattered across NJ tree (Fig. 4). This pattern of evolution suggests that *gma-pre-MIR394* sequences arose from multiple duplication events, indicating that there has been a prevalence of higher rate of duplication in the *Glycine max* genome [35].

Exceptionally, we found one sequence *atr-pre-MIR394*, from species *Amborella trichopoda* was placed with another dicot *pre-MIR394s* in cluster II (Fig. 4). The studies related to evolutionary relationship of *Amborella trichopoda* with other angiosperms have categorized *Amborella trichopoda* as the sole surviving sister species of all other angiosperms [36, 37]. The emergence and evolution of *MIR394* gene is observed in the basal angiosperms, at the very beginning of gymnosperm-angiosperm divergence prompting us to suggest that *MIR394* genes have evolved exclusively in angiosperms.

The phylogenetic analysis of mature and precursor sequences has shown that the mature ones are more conserved than their precursors. The *pre-MIR394s* are of longer length than the mature miR394s, making them more vulnerable to mutation/s or other nucleotide changes during evolution causing significant sequence variation. Therefore, we observed random distribution of *pre-MIR394* sequences with varied rate of distribution, showing extremely less conservation amongst them. This is evident from the distribution pattern of the highly conserved cca-miR394, ssl-miR394, era-miR394 and rsa-miR394 in the ML tree (Fig. 3, Tables 1 and 2), while their *pre-MIR394s* are separated distantly with the longest branch lengths, signifying their faster evolution with the highest rate of substitution (Fig. 4).

The targets of miR394s are often conserved divulging its functionality in significant physiological and developmental processes. Thus, to decipher the functional conservation/diversification of miR394, we predicted the targets and reconstructed a phylogenetic tree (Fig. 5). Prior to the phylogenetic analysis, we clustered the mature miR394 sequences using MSA to identify the UmiR394 sequences (Fig. 1). Further, these UmiR394 sequences were used for the identification of their targets and complementary binding sites known as UTSs (Table 3). Sixteen UTSs for eight UmiR394s were identified, targeting forty-three different genes. Most of these target genes belonged to the *F-box* protein encoding genes (Table 3). Subsequently, the identified UTSs and predicted targets were used for the reconstruction of an unrooted ML tree [10]. The resultant ML tree was divided into many clades (Fig. 5). The UmiR394–1 and -2 contained all the miR394/mi394–5p members and rest of UmiR394s have miR394–3ps (Table 3). We observed that the UTSs 1a, 1e, 1f, 1 k and 2 are closely related with all the targets which were either *F-box* or unannotated *F-box* genes (Fig. 5). Other 12 UTSs were found to be closely related to target gene not belonging to *F-box*, and forming a separate individual clade (Fig. 5). Out of eleven UTSs, four UTSs (UTS 3, 4, 5 and 8) have UmiR394s which consisted of miR394–3ps, which have non-conserved target (Table 3). As evident from the MSA, the miR394–3ps have variation in their sequences (Fig. 1), which resulted into a separate cluster of miR394–3ps in phylogenetic tree (Fig. 3). Hence the functional deviation of miR394–3ps was attributed to its 3′ end (anti-sense) processing from precursor sequences causing significant sequence variation. Though non-conserved targets were also identified, our analysis suggest that *F-box* genes are highly conserved targets of miR394s. The functional diversification of miRNAs by acquiring new targets are due to nucleotide variation in the mature miRNA sequence. The genes may also gain/alter miRNA target sites either by mutations or via segmental duplication, speciation or climatic adaptation [11, 38–40]. Some of the miRNAs such as miR167 are reported to cleave non-conserved targets *IAA-ALANINE RESISTANT 3* (*IAR3*) in *Arabidopsis* [38] and *CNBL10* in apple [10]. Similarly, miR827 is also known to targets two different genes, *NLA* and *PHT5* in *Arabidopsis* and rice, respectively [39]. Extensive analysis of the unannotated paralog *F-box* genes across the plant kingdom is further required for a better understanding of its evolutionary significance as well as target preference.

## Conclusions

We have identified 79 miR394s, including 20 novel miRNAs, and their target *LCR* homologs from selected and newly sequenced plant species. The phylogenetic analysis highlights the co-evolutionary pattern of miR394s and their conserved/diverged targets. We observed that the miR394s/miR394–5ps are highly conserved among plants species irrespective of their less conserved *MIR394s.* Though, miR394–3ps were complementary to miR394s/miR394–5ps, they clustered separately in the phylogeny suggesting their independent occurrence and a possible justification for their functional diversification. The phylogenetic analysis also suggested that the miR394s had evolved at the beginning of gymnosperm-angiosperm divergence. Further, we identified and computationally validated 20 novel miR394s from 14 newly sequenced genomes using our annotation workflow. *LCRs*, the member of *F-box* protein coding genes and conserved target of miR394, were also found to be conserved across different plant species. The phylogeny of miR394 targets and their complementary binding sites have suggested their conservation. Some functional diversification was observed since few of the miR394s were targeting non-conserved targets. Future studies are needed to understand the functional relevance of the selection of non-conserved targets of miR394 in different species.

## Methods

### Identification of miR394s and their precursor sequences

To identify the number of miR394 sequences available, we used the miRNA registry database (miRBase version 21, http://www.mirbase.org/). The key word “miR394” was used as query against miRBase to search the miR394 family members in each plant species. Fifty-nine mature miR394 and their precursor *MIR394* sequences from various plant species were retrieved. We did not find miR394 from any species other than angiosperms (Table 1). The nomenclature for species used for this study is in accordance with miRBase such as for *Arabidopsis thaliana* as “ath” (Table 1). The miR394 entries in miRBase were further verified using BLAST search in NCBI, (http://www.ncbi.nlm.nih.gov/), and other species-specific databases. For phylogenetic analysis, only miRBase registered miR394 were considered and used.

While searching NCBI database, we found some ncRNA sequences having 100% identity with reference miR394 from newly sequenced genomes. These ncRNA sequences were further scanned using the tools tRNAscan-SE 2.0 (http://lowelab.ucsc.edu/tRNAscan-SE/) and RNAmmer 1.2 server (http://www.cbs.dtu.dk/services/RNAmmer/) for ruling out as t-RNA and r-RNA, respectively [41, 42]. Rest of the ncRNA gene sequences were subjected to the identification of canonical precursor stem-loop structures and in silico validation through publicly available online tools miRNAFold (https://evryrna.ibisc.univ-evry.fr/evryrna/mirnafold) and [43, 44] RNAfold web server (http://rna.tbi.univie.ac.at/cgi-bin/RNAWebSuite/RNAfold.cgi), a core program of the Vienna RNA package [45]. For the identification of precursor sequences from ncRNA genes miRNAFold was used. The parameters were set as default with the precursor length set at ≤170 nt. The potential precursors containing miR394 sequences were used for the prediction of secondary structures (stem-loop structure) through RNAfold web server. The RNAfold webserver calculates minimum free energy (MFE) of predicted secondary structure of RNAs using the dynamic programming algorithm [46] and equilibrium base-pairing probabilities are calculated through John McCaskill’s partition function (PF) algorithm [47]. The secondary structures were chosen from those having ≥18 bp of mature miR394 matching in the folded region followed by a central loop (20 novel miR394s from 14 newly sequenced genomes). Further, each predicted precursor *MIR394* sequences from the newly sequenced genome was manually curated and named according to species as used in miRBase database (Table 2).

### Phylogenetic analysis and target prediction from unique identical miR394 sequences

The retrieved miR394 sequences from all the species were aligned together using ClustalX2, and on the basis of this alignment unique identical sequences were identified (Fig. 1, Table 2). Further these unique identical sequences of miR394 were used for target prediction. The statistical significance of sequence similarity/identity of aligned miR394 as well as precursor sequences were calculated through Kolmogorov-Smirnov statistical test in GeneDoc v2.7.0 (summarized in Additional file 1; Figure S1) [48]. Further aligned miR394 sequences were used for studying their comparative evolutionary process by generating phylogenetic tree through MEGA6 [49]. The parameters used for the maximum likelihood (ML) method to generate unrooted tree by considering all the sites in the mature sequences were same as described previously [9, 10]. The ML tree was reconstructed by taking gamma distribution with Invariant sites (G + I) for rate calculation having discrete Gamma distribution among sites (4 categories) and GTR Substitution model. The bootstrap calculated for 1000 replicates. The ML tree was inferred through BioNJ as the initial tree using heuristic method [9, 10]. Additionally, *pre-MIR394s* were used for the phylogenetic analysis by reconstructing the NJ tree using MEGA6 [10]. The unrooted NJ tree was reconstructed by taking partial deletion with uniform rates and using Maximum Composite Likelihood model. The bootstrap was also calculated for 1000 replicates. The targets of uniquely clustered miR394 sequences (Table 2) were predicted either by *psRNATarget* tool or BLAST from NCBI. Consecutively, these target genes were also used for phylogenetic analysis with MEGA6. Since the target sequences did not show any consensus during multiple sequence alignment, distance estimation (distance matrix) method was used. Further, the substitution model, p-distance with uniform rates, and pairwise deletion for the gap treatment were used for the reconstruction of phylogenetic tree.

The unique identical miR394 (UmiR394) sequences were used for the prediction of targets using *psRNATarget* tool [50]. The analysis parameters, maximum E-value and UPE score (maximum energy to unpair the target site) were chosen as 5 and 50, respectively. The complementary binding sequences UTSs of UmiR394 were identified and numbered (Table 2). These identified UTS were selected on the basis of lowest E-value and UPE score and numbered (as 1, 2, 3 etc.; Table 3). Though, UTS and their corresponding target genes were predicted by *psRNATarget* tool, some of UmiR394s did not contain any predicted UTS. Therefore, putative target genes were searched for these UmiR394s using NCBI BLAST (http://blast.ncbi.nlm.nih.gov/Blast.cgi) keeping 100% query coverage and identity, in all available plant genomes. The nomenclature for the target genes used in this study was specified here as “Ath-LCR” to indicate *A. thaliana LCR*. Same format was used for target genes from other species (Table 3). Further, these miR394 targets and UTS were used for phylogenetic analysis to understand the evolutionary pattern.

## Additional file

Additional file 1: **Figure S1.** Statistical analysis using Kolmogorov-Smirnov test through GeneDoc v2.7.0. Calculation of percent identity of aligned miR394 sequences for statistical significance. A. The test has shown that the ≥0.3 fraction of mature miR394 sequences have ≥90% sequence identity B. ~ 0.2 fraction of the precursor *pre-MIR394* sequences have > 55% sequence identity. **Figure S2.** An unrooted ML phylogeny of miR394s along with reverse complementary sequences of miR394–3p using MEGA6. This tree contains reverse complementary sequences of miR394–3p (highlighted, encircled portion). All miRNA394/miR394 − 5 ps are highly conserved with very less substitution rate. The scale bar represents the nucleotide substitution rate. (ZIP 377 kb)

## Abbreviations

Hypo: Hypothetical
miR394–3pRC: Reverse complementary sequences of miR394–3p
miR394s/miR394–5p: miR394 sequences which were processed from 5′ end of stem loop
UmiR394: Unique miR394
Unch: Uncharacterized
UTS: Unique Target Sequence
