# Supplementary figures and images for: Identification and co-evolution pattern of stem cell regulator miR394s and their targets among diverse plant species
Source: BMC Evol Biol. 2019 Feb 14;19:55. doi: 10.1186/s12862-019-1382-7 (PMC6376759; doi:10.1186/s12862-019-1382-7)

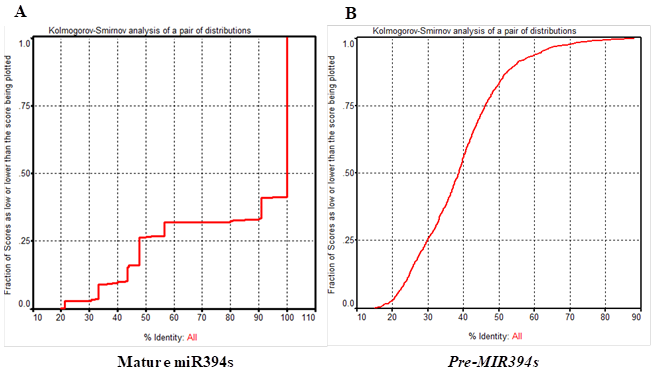

Supplement: Supplementary file 1 — Figure S1. Statistical analysis using Kolmogorov-Smirnov test through GeneDoc v2.7.0. Calculation of percent identity of aligned miR394 sequences for statistical significance. A. The test has shown that the ≥0.3 fraction of mature miR394 sequences have ≥90% sequence identity B. ~ 0.2 fraction of the precursor pre-MIR394 sequences have > 55% sequence identity. Figure S2. An unrooted ML phylogeny of miR394s along with reverse complementary sequences of miR394–3p using MEGA6. This tree contains reverse complementary sequences of miR394–3p (highlighted, encircled portion). All miRNA394/miR394 − 5 ps are highly conserved with very less substitution rate. The scale bar represents the nucleotide substitution rate. (ZIP 377 kb) [file 12862_2019_1382_MOESM1_ESM.zip › Fig S1.TIF]

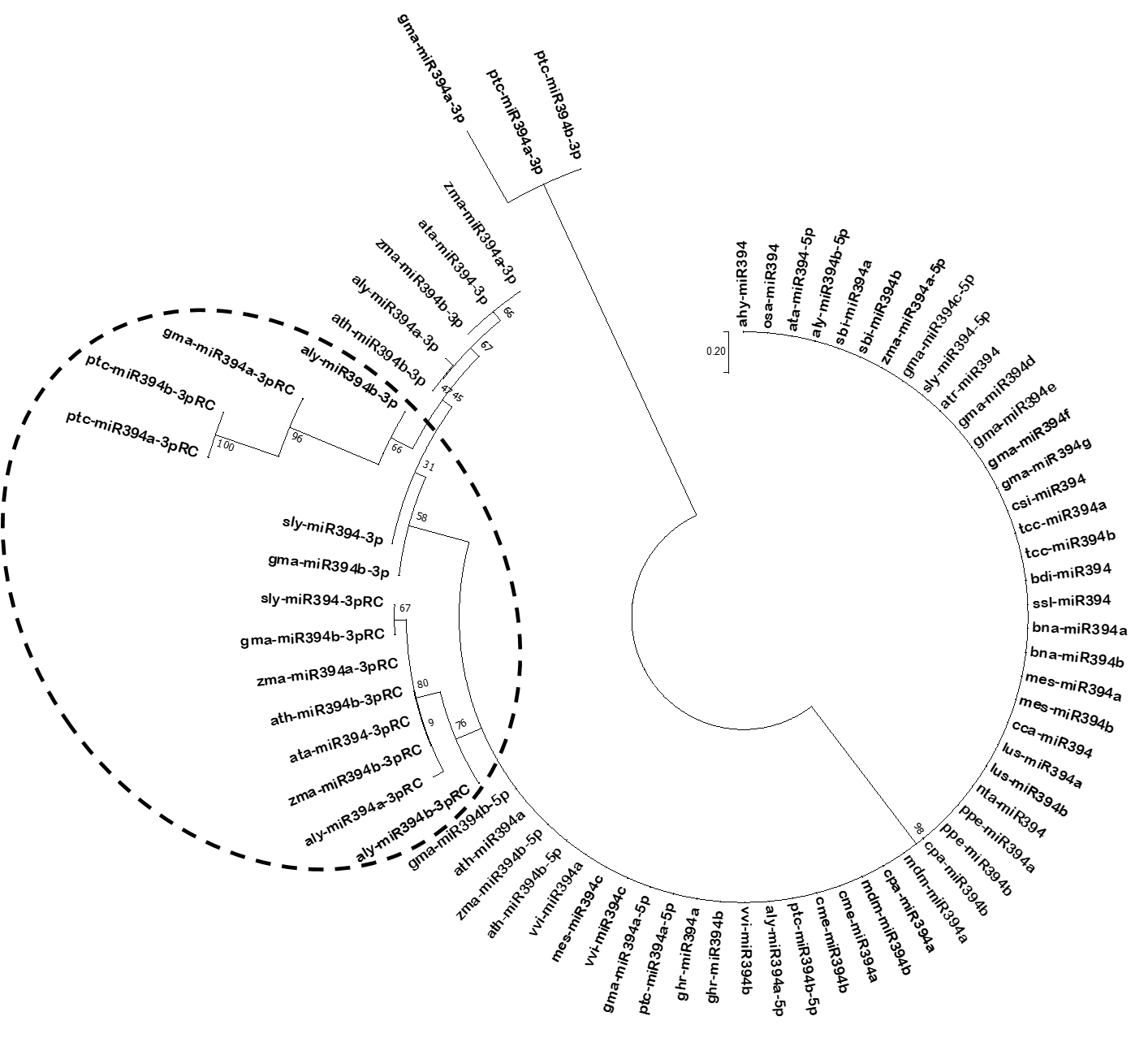

Supplement: Supplementary file 1 — Figure S1. Statistical analysis using Kolmogorov-Smirnov test through GeneDoc v2.7.0. Calculation of percent identity of aligned miR394 sequences for statistical significance. A. The test has shown that the ≥0.3 fraction of mature miR394 sequences have ≥90% sequence identity B. ~ 0.2 fraction of the precursor pre-MIR394 sequences have > 55% sequence identity. Figure S2. An unrooted ML phylogeny of miR394s along with reverse complementary sequences of miR394–3p using MEGA6. This tree contains reverse complementary sequences of miR394–3p (highlighted, encircled portion). All miRNA394/miR394 − 5 ps are highly conserved with very less substitution rate. The scale bar represents the nucleotide substitution rate. (ZIP 377 kb) [file 12862_2019_1382_MOESM1_ESM.zip › Fig S2.tif]
